# TooT-T: discrimination of transport proteins from non-transport proteins

**DOI:** 10.1186/s12859-019-3311-6

**Published:** 2020-04-23

**Authors:** Munira Alballa, Gregory Butler

**Affiliations:** 1 Department of Computer Science and Software Engineering, Concordia University, Montréal, Québec, Canada; 2 Centre for Structural and Functional Genomics, Concordia University, Montréal, Québec, 24105 Canada

**Keywords:** Transporter prediction, Ensemble learning, Amino acid composition

## Abstract

**Background:**

Membrane transport proteins (transporters) play an essential role in every living cell by transporting hydrophilic molecules across the hydrophobic membranes. While the sequences of many membrane proteins are known, their structure and function is still not well characterized and understood, owing to the immense effort needed to characterize them. Therefore, there is a need for advanced computational techniques takes sequence information alone to distinguish membrane transporter proteins; this can then be used to direct new experiments and give a hint about the function of a protein.

**Results:**

This work proposes an ensemble classifier *TooT-T* that is trained to optimally combine the predictions from homology annotation transfer and machine-learning methods to determine the final prediction. Experimental results obtained by cross-validation and independent testing show that combining the two approaches is more beneficial than employing only one.

**Conclusion:**

The proposed model outperforms all of the state-of-the-art methods that rely on the protein sequence alone, with respect to accuracy and MCC. TooT-T achieved an overall accuracy of 90.07% and 92.22% and an MCC 0.80 and 0.82 with the training and independent datasets, respectively.

## Background

Membrane transport proteins control the movement of molecules across the membrane so that essential molecules such as sugars and amino acids enter the cell while waste compounds leave the cell. It is estimated that membrane transport proteins encode 2% to 16% of open reading frames in prokaryotic and eukaryotic genomes, highlighting the importance of transporters in all living species [1]. Any defective or mis-regulated membrane proteins can disturb the body’s homoeostasis, thereby causing disease. Therefore, the study of cell membranes is critical for understanding the causes of many diseases and determining how to treat them. Membrane proteins are exceptionally attractive targets for the pharmaceutical industry. Indeed, over half of today’s FDA-approved drugs target them [2].

While many sequences of membrane proteins are known, due to the large number of recent genome projects, their structures and functions remain poorly characterized and understood. This is related to the immense effort necessary to characterize them because of their flexibility and instability, which creates challenges at many levels, including crystallization, expression, and structure solution. This unbalanced reality between the number of available sequences and the experimentally characterized ones has created many obstacles in the advancement of biology and drug discovery. Therefore, there is a need for advanced computational techniques that take sequence information alone to distinguish membrane transporter proteins; this can then be used to direct new experiments and offer clues about the function of a protein.

Earlier efforts applied homology searches of experimentally characterized databases to detect novel transporters, homology searches are still commonly used by many tools. For example, *TransATH* [3] (Transporters via Annotation Transfer by Homology) is a system that automates Saier’s protocol based on sequence similarity. *TransATH* includes the computation of subcellular localization and improves the computation of transmembrane segments. The parameters of TransATH are chosen for optimal performance based on a gold standard set of transporters and non-transporters from *S. cerevisiae*. *TransATH* reports an overall accuracy of 71.0%. In addition, Barghash et al. [4] annotated transporters at family and substrate levels from three organisms using sequence similarity and sequence motifs. A major limitation of homology methods, however, is that they can generate false assignments because homologous sequences do not always have significant sequence similarities. Likewise, proteins with high sequence similarities do not always share the same function [5].

More advanced methods attempt to overcome the limitations of homology methods by utilizing features from the protein sequences that better reflect the relation between the sequences and the target function. For example, *TrSSP* (Transporter Substrate Specificity Prediction Server) [6] is a web server for predicting membrane transport proteins and their substrate category. The *TrSSP* tool applies SVM in combination with the Amino Acid index (AAindex) and Position-Specific Scoring Matrix (PSSM) to predict top-level transporters and achieves a transporter prediction accuracy of 78.99% and 80.00% and a Matthews correlation coefficient (MCC) of 0.58 and 0.57 during the cross-validation and the independent testing, respectively.

*SCMMTP* [7] uses a novel scoring card method (SCM) that utilizes dipeptide composition to identify putative membrane transport proteins. The *SCMMTP* method first builds an initial matrix of 400 dipeptides and uses the difference between positive and negative compositions as an initial dipeptide scoring matrix. This matrix is then optimized using a genetic algorithm. *SCMMTP* achieved an overall accuracy of 81.12% and 76.11% and an MCC of 0.62 and 0.47 with the training and independent datasets, respectively.

Li et al. [8] uses SVM to predict substrate classes of transmembrane transport proteins by integrating features from PSSM, amino acid composition, biochemical properties, and Gene Ontology (GO) terms. They achieved an overall accuracy of 98.33% and an MCC of 0.97 with the independent dataset. Their method incorporates the GO annotation as a feature that is likely to be missing in non-annotated sequences.

Ou et al. [9] applies a word-embedding natural language processing approach to protein sequences of transporters. The protein sequence is defined as using both the word embedding and frequencies of its biological words. They achieved outstanding substrate specificity for the transporters but not for transporter detection. The accuracy for transporter prediction only reached 83.94% during the cross-validation and 85.00% with the independent datasets.

The findings from previous studies on transporter prediction can be summarized as follows: Support Vector Machine (SVM) shows superior performance compared to other machine-learning algorithms [7–9]. Moreover, the PSSM profile is a highly accurate feature for demonstrating the evolutionary information in protein sequence functional classification [6, 7, 10].

This work focuses on distinguishing membrane transporter proteins from other non-transporter proteins. The main contributions of this work can be summarized as follows:

- We explore the practicality of using traditional homology search techniques to detect transporter proteins.
- We compare the performance of various discriminators/features on SVM models and introduce a new feature, called *psi-composition*, which shows superior performance to all other examined features.
- We propose a new tool, *TooT-T*, which employs an ensemble classifier that is trained to optimally combine the predictions obtained from homology annotation transfer and psi-composition based models to determine the final prediction. The ensemble exploits the low correlation between the predictions obtained by various methods to build a more robust classifier. The proposed model outperforms all of the state-of-the-art methods that rely on the protein sequence alone, with an overall accuracy of 90.07% and 92.22% and an MCC on 0.80 and 0.82 for the training and independent datasets respectively.

## Methods

### Overview

**Fig. 1:**
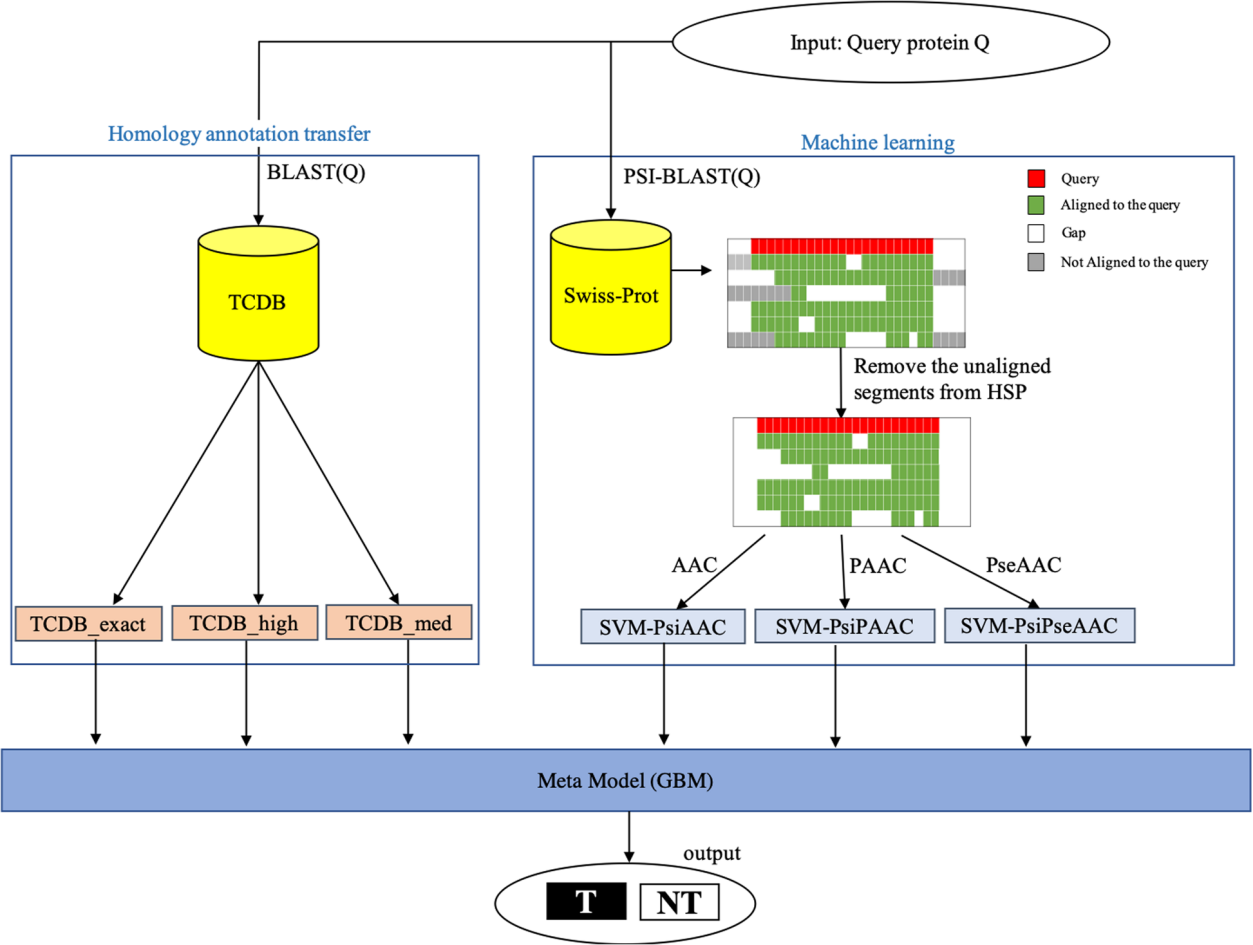
TooT-T overview

We propose an ensemble classifier that combines the results generated by two distinct methods, namely homology annotation transfer and machine learning, to detect transporter proteins. First, given a query protein Q, a traditional homology search of the Transporter Classification Database (TCDB) is performed utilizing BLAST. A query is predicted as transporter if a hit is found using three predetermined sets of thresholds. The three predictions are delivered into the ensemble. Then, three variations of psi-composition features —psiAAC, psiPAAC, and psiPseAAC— are computed and input into their respective trained SVM models, the subsequent predictions are delivered to the ensemble. Finally, the trained ensemble meta-model predicts the final class as transporter *T* or non-transporter *NT*. Figure 1 delineates an overview of the prediction steps. Detailed descriptions of each step are presented in the following sections.

### Dataset

The same benchmarking dataset used by most transporter predictors, such as *TrSSP* [6], *SCMMTP* [7], Li et al. [8], and Ou et al. [9], was used to build this system.

This benchmarking dataset provided by Mishra *et al.* (available at http://bioinfo.noble.org/TrSSP/?dowhat=Datasets) is collected from the Swiss-Prot database. The dataset initially contained 10,780 transporter, carrier, and channel proteins that were well characterized at the protein level and had clear substrate annotations. Then, Mishra *et al.* removed the transporters with more than two substrate specificities, sequences with biological function annotations based solely on sequence similarity, and sequences with greater than 70% similarity. The final dataset of Mishra *et al.* contained a total of 1,560 sequences, divided into training and test sets, as presented in Table 1.

**Table 1:** The dataset

Class	Training dataset	Testing dataset
Transporter	780	120
Non-Transporter	600	60
Total	1380	180

### Position specific iterated alignment compositions

The PSI-BLAST [11] (3 iterations, e-value cutoff 0.001) search was performed on a sample protein sequence using a modified version of the Swiss-Prot database (release 2018_6) to find homologous sequences. The modified Swiss-Prot database does not include the exact hits of test sequences. Regions in the database hit sequences that were not aligned with the query protein were discarded. The query protein (Q) and the aligned regions of its hits (*h*_1_,*h*_2_,...,*h*_*n*_) were then used to compute position-specific iterated amino acid composition (psiAAC), pair amino acid composition (psiPAAC), and pseudo amino acid composition (psiPseAAC) as follows:

#### Position Specific Iterated Amino Acid Composition (psiAAC)

The AAC of the query protein (Q) and each of its filtered hits (*h*_1_,*h*_2_,…,*h*_*n*_) were calculated separately as the fractions of all 20 natural amino acids and as:

1 $$ c_{i} = \frac{F_{i}}{L} \qquad i=(1,2,3,...20)  $$

where *F*_*i*_ is the frequency of the *i*^*th*^ amino acid and *L* is the length of the sequence. The AAC is represented as a vector of size 20:

2 $$ AAC(P_{x}) \,=\, \left [ c_{1}, c_{2}, c_{3},..., c_{20} \right] \qquad x \in (Q, h_{1},h_{2} \dots, h_{n})  $$

where *c*_*i*_ is the composition of *i*^*th*^ amino acid. The mean of individual AAC compositions represents the psiAAC for Q and was computed as:

3 $$ {\begin{aligned} {AAC}_{psi}(Q) &= \frac{1}{n+1}\sum{ AAC(P_{x})} \\&\qquad x \in (Q, h_{1},h_{2} \dots, h_{n}) \end{aligned}}  $$

#### Position Specific Iterated Pair Amino Acid Composition (psiPAAC)

Similarly, the individual PAAC descriptors for the query protein (Q) and each of its filtered hits (*h*_1_,*h*_2_,…,*h*_*n*_) were calculated as

4 $$ d_{i,j} = \frac{F_{i,j}}{L-1} \qquad i,j=(1,2,3,...20)  $$

where *F*_*i*,*j*_ is the frequency of the *i*^*th*^ and *j*^*th*^ amino acids as a pair (dipeptide) and *L* is the length of the sequence. Like AAC, PAAC is represented as a vector of size 400, as follows:

5 $$ {\begin{aligned} PAAC(P_{x}) &= \left [ d_{1,1}, d_{1,2}, d_{1,3},..., d_{20,20} \right] \qquad \\&x \in (Q, h_{1},h_{2} \dots, h_{n}) \end{aligned}}  $$

where *d*_*i*,*j*_ is the dipeptide composition of the *i*^*th*^ and *j*^*th*^ amino acid. The mean of individual PAAC compositions represents the psiPAAC for Q and was computed as:

6 $$ {\begin{aligned} {PAAC}_{psi}(Q) &= \frac{1}{n+1}\sum{ PAAC(P_{x})} \\& \qquad x \in (Q, h_{1},h_{2} \dots, h_{n}) \end{aligned}}  $$

#### Position Specific Iterated Pseudo Amino Acid Composition (psiPseAAC)

The PseAAC is a combination of the 20 components of the conventional amino acid composition and a set of sequence order correlation factors that incorporates certain biochemical properties, originally proposed by Chou [12]. Given a protein sequence of length *L*:

7 $$ R_{1} R_{2} R_{3} R_{4}... R_{L}  $$

a set of descriptors called sequence order-correlated factors are defined as:

8 $$ \left \{ \begin{array}{c} \theta_{1} = \frac{1}{L-1} \sum_{i=1}^{L-1} \Theta (R_{i},R_{i+1}) \\ \smallskip \theta_{2} = \frac{1}{L-2} \sum_{i=1}^{L-2} \Theta (R_{i},R_{i+2}) \\ \smallskip \theta_{3} = \frac{1}{L-3} \sum_{i=1}^{L-3} \Theta (R_{i},R_{i+3}) \\.\\.\\.\\ \theta_{\lambda} = \frac{1}{L-\lambda} \sum_{i=1}^{L-\lambda} \Theta (R_{i},R_{i+\lambda}) \\ \end{array} \right.  $$

The parameter *λ* is chosen such that (*λ*<*L*). A correlation function is given by:

9 $$ {{}\begin{aligned} \Theta (R_{i},R_{j})= \frac{1}{3} \left \{ [ H_{1}(R_{j}) - H_{1}(R_{i}) ]^{2} +[ H_{2}(R_{j}) - H_{2}(R_{i}) ]^{2} \right. \\ \left. + [ M(R_{j}) - M(R_{i}) ]^{2} \right\} \end{aligned}}  $$

where *H*_1_(*R*) is the hydrophobicity value, *H*_2_(*R*) is hydrophilicity value, and *M*(*R*) is side chain mass of the amino acid *R*_*i*_. Those quantities were converted from the original hydrophobicity, original hydrophilicity, and original side chain mass values by standard conversion as follows:

10 $$ H_{1} (R_{i}) = \frac{H^{\circ}_{1} (R_{i}) - \frac{1}{20} \sum_{k=1}^{20} H^{\circ}_{1} (R_{k})} {\sqrt { \frac { \sum_{y=1}^{20} \left[ H^{\circ}_{1} (R_{y}) - \frac{1}{20} \sum_{k=1}^{20} H^{\circ}_{1} (R_{k}) \right]^{2}} {20}} }  $$

where *H*1∘(*R*_*i*_) is the original hydrophobicity value for the amino acid *R*_*i*_ that was taken from Tanford [13]; *H*2∘(*R*_*i*_) and *M*^∘^(*R*_*i*_) are converted to *H*_2_(*R*_*i*_) and *M*(*R*_*i*_) in the same way. The original hydrophilicity value *H*2∘(*R*_*i*_) for the amino acid *R*_*i*_ was taken from Hopp and Woods [14]. The mass *M*^∘^(*R*_*i*_) of the *R*_*i*_ amino acid side chain can be obtained from any biochemistry textbook. PseAAC is represented as a vector of size (20+*λ*) as follows:

11 $$ {\begin{aligned} PseAAC(P_{x}) &= \left [s_{1},..., s_{20}, s_{21},...,s_{20+ \lambda} \right]\\& \qquad x \in (Q, h_{1},h_{2} \dots, h_{n}) \end{aligned}}  $$

where *s*_*i*_ is the pseudo-amino acid composition such that:

12 $$ s_{i} = \left \{ \begin{array}{cc} \frac{f_{i}}{\sum_{r=1}^{20} f_{r}+ \omega \sum_{j=1}^{\lambda} \theta_{j}} & 1 \leq i \leq 20 \\ \\ \frac{\omega \theta_{i-20}}{\sum_{r=1}^{20} f_{r}+ \omega \sum_{j=1}^{\lambda} \theta_{j}} & 20 < i \leq 20 + \lambda \end{array} \right.  $$

where *f*_*i*_ is the normalized occurrence frequency of the of the *ith* amino acid in the protein sequence, *θ*_*j*_ is the *j*^*th*^ sequence order-correlated factor calculated from Equation 8, and *ω* is a weight factor for the sequence order effect. The weight factor *ω* puts weight on the additional PseAAC components with respect to the conventional AAC components. The user can select any value from 0.05 to 0.7 for the weight factor. The default value given by Chou [12] is.05.

The mean of individual PseAAC compositions represents the psiPseAAC for Q and was computed as follows:

13 $$ {\begin{aligned} {PseAAC}_{psi}(Q) &= \frac{1}{n+1}\sum{ PseAAC(P_{x})}\\& \qquad x \in (Q, h_{1},h_{2} \dots, h_{n}) \end{aligned}}  $$

### Support-vector machine

The SVM is a powerful machine-learning tool that is used in many biological prediction tools, such as [6] and [9]. We used SVM with an RBF kernel as implemented by R e1071 library version 1.6-8. The best combination of C and *γ* parameters was determined utilizing a grid-search approach.

### Annotation transfer by homology

Unlike the discrete representation of a protein sample in the psi-compositions, here the protein sample was represented by its amino acid sequence and used in a similarity search-based tool (BLAST) to find similar matches in the TCDB [15]. The TCDB uses the classification system approved by the International Union of Biochemistry and Molecular Biology (IUBMB) for membrane transport proteins, known as the transporter classification (TC) system. The TCDB is a curated database of accurate and experimentally characterized transporters from over 10,000 published references. If the BLAST search produced a hit, the query was predicted to be a transporter. Since applied thresholds play an essential role in the quality of prediction, different thresholds were utilized, as shown in Table 2.

**Table 2:** Different Blast thresholds on TCDB

Name	BLAST Threshold	Motivation
TCDB_exact	e-value=0; percent identity 100%	exact match
TCDB_high	e-value 1e–20; percent identity 40%; query coverage 70%; subject coverage 70%; and difference in length of 10%	thresholds recommended by Butler et al. [3] for TCDB Blast
TCDB_med	e-value 1e–8%	threshold recommended by Barghash et al. [4] as an acceptable normalized BLAST threshold when dealing with a TC system

### Ensemble classifier

We applied an ensemble technique known as stacked generalization, or stacking [16] to develop TooT-T. Instead of combining the predictions from multiple predictors using a simple function (such as voting), stacking trains a new model to perform the aggregation.

The stacking framework involves two levels of learning. The first level contains *base-classifiers* that learns directly from the training data. The second level contains a *meta-classifier*, that is trained using the predictions from the base-classifiers. The training instances of the meta-classifier were generated while performing the cross-validation. Algorithm 1 illustrates how the training dataset of the meta-classifier is generated [17].


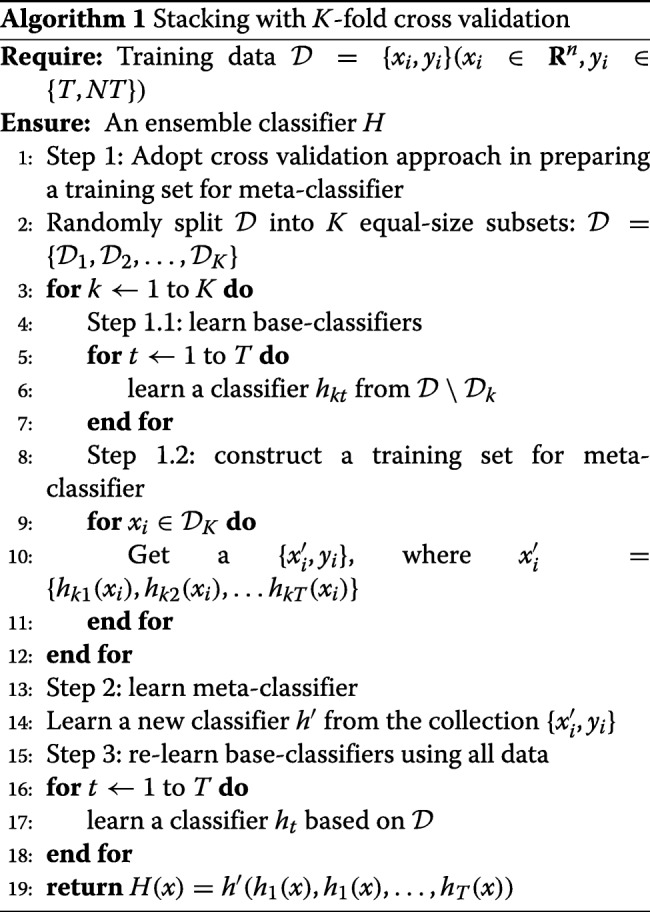


When a new query protein is input into TooT-T, the class of the query is predicted by the six base classifiers: three from SVM models that use psiAAC, psiPAAC, and psiPseAAC features respectively, and three using annotation transfer by homology utilizing different thresholds: TCDB_exact, TCDB_high, and TCDB_med. The six predictions are then input into the meta-classifier, which outputs the final prediction. The Gradient Boosting Machine (GBM), as implemented by *caret* package in R, was utilized to develop the meta-classifier.

### Performance evaluation

The performance of different models was evaluated on the training dataset using 10-fold cross-validation (10-CV), in which the training dataset was randomly partitioned into ten equally sized sets. A single set was kept as the validation data, and the remaining nine sets were used to train the respective model. The trained model was then tested using the validation set. The cross-validation process was repeated ten times, and each of the sets was used once as the validation data. The performance of each model was averaged to produce a single estimation. Since the 10-fold performance varies with different random splits, and to make the error estimation more stable, we repeated the 10-CV ten times with different random partitions, and the performance variations between runs were captured by computing the standard deviation. It has been reported [18] that the repeated version stabilizes the error estimation, and therefore it reduces the variance of the k-cv estimator. Throughout the rest of the paper, the cross-validation performance is reported as *mean*±*sd* of the ten different runs of the 10-CV.

Furthermore, the independent dataset was also used to perform a thorough evaluation experiment. The data in the independent dataset were not used during the training process and are completely unknown to our models. Four main evaluation metrics are were used to evaluate the performance: sensitivity, specificity, accuracy, and the MCC. Sensitivity, which calculates the proportion of positives (transporters) that are correctly identified.

14 $$ Sensitivity = \frac{TP}{TP+FN}  $$

Specificity, which measures the proportion of non-transporters that are correctly identified.

15 $$ Specificity = \frac{TN}{TN+FP}  $$

Accuracy, which refers to the proportion of correct predictions made divided by the total number of predictions.

16 $$ Accuracy = \frac{TP+TN}{TP+FN+TN+FP}  $$

The MCC is less influenced by imbalanced tests because it takes into account true and false positives and negatives. MCC values range from 1 to −1, where 1 indicates a perfect prediction, 0 represents no better than random, and −1 implies total disagreement between prediction and observation. Higher MCC values mean that the predictor has high accuracy with positive and negative classes as well as less misclassification with the two classes. MCC is considered to be the best singular assessment metric when the data are imbalanced [19–21].

17 $$ {{}\begin{aligned} MCC = \frac{(TP\times TN - FP \times FN)} { \sqrt{ (TP + FP) \times (TP + FN) \times (TN + FP) \times (TN + FN) }} \end{aligned}}  $$

## Results and discussion

### Performance of transporter classification of different features

The goal is to find the most discriminative features to represent a protein sequence, Table 3 presents the cross-validation performance of various features on SVM models.

**Table 3:** Average performance of different models

	Name	Sensitivity (%)	Specificity (%)	Accuracy (%)	MCC
SVM	psiPAAC*	86.73 ±0.29	87.99 ±0.54	87.29 ±0.11	0.7448 ±0.0027
	blast-PAAC	87.03 ±0.37	86.08 ±0.24	86.62 ±0.22	0.7299 ±0.0045
	psiAAC*	82.69 ±0.21	90.64 ±0.41	86.13 ±0.15	0.7278 ±0.0036
	psiPseAAC*	80.18 ±0.58	91.51 ±0.45	85.13 ±0.40	0.7125 ±0.0075
	blast-AAC	84.97 ±0.35	84.14 ±0.52	84.61 ±0.22	0.6897 ±0.0050
	PSSM	83.83 ±0.59	82.03 ±0.59	83.06 ±0.21	0.6579 ±0.0038
	blast-PseAAC	84.59 ±0.53	78.19 ±0.82	81.81 ±0.35	0.6306 ±0.0077
	PseAAC	80.45 ±0.42	70.62 ±0.70	76.19 ±0.44	0.5149 ±0.0098
	AAC	79.73 ±0.50	70.66 ±0.89	75.79 ±0.51	0.5069 ±0.0101
	PAAC	77.93 ±0.31	72.14 ±0.56	75.41 ±0.31	0.5014 ±0.0062

The table shows *mean*±*sd* performance of ten different runs of the 10-CV, in ascending order of accuracy. The asterisk symbol (*) refers to the features used in TooT-T

The examined features include: the baseline compositions where no evolutionary information is incorporated (AAC, PAAC, PseAAC), the commonly used feature to encode evolutionary information PSSM (implemented as in [6] using the same psi-composition thresholds (3 iterations, e-value cutoff 0.001)), compositions computed from sequences retrieved from the BLAST search (blast-AAC, blast-PAAC, blast-PseAAC) (e-value cutoff 0.001), and the proposed features (psiAAC, psiPAAC, psiPseAAC). Since the training data is balanced, we focus on the accuracy to evaluate the performance of different models.

The baseline compositions do not exhibit great variation in performance and have an average accuracy of 75.80%. The accuracy is further boosted when evolutionary information is incorporated. While PSSM is most commonly applied in the literature to encode evolutionary information, we find that in most cases features that combine amino acid composition with evolutionary information (as described in the “Methods” section) yield higher accuracy for transporter prediction. Since the PSSM feature is also extracted from PSI-BLAST output, it is expected to show an improved performance to at least the BLAST-compositions, but this is not what is portrayed by our results. One explanation for this could be that the commonly used PSSM feature is computed from the original PSSM profile output from PSI-BLAST search to make it fixed in size 20×20. The PSSM feature, although superior to the baseline, does not capture properties to the extent shown by the amino acid composition on the returned sequences. Among all tested features, psiPAAC obtained the highest accuracy of 87.29%.

The high performance of the psi-composition features is a result of incorporating two distinctive approaches, namely amino acid composition and evolutionary information. The idea is that multiple homologous sequences can reveal more about the function of a protein than a single sequence. Homologous sequences can be inferred when they share more similarity than would be expected by chance [22]. Similarity tools such as BLAST help to minimize false positives (non-homologs with significant scores; Type I errors) but do not necessarily detect remote homologs (homologs with non-significant scores; Type II errors) [22]. PSI-BLAST is more sensitive in terms of finding such remote homologs, and thus utilized by the proposed features. Furthermore, the alignment results of PSI-BLAST contains valuable information about the most conserved regions in the protein, such conservation can reflect the function of the protein. Computing the average amino acid composition from the aligned homologous sequences thus provides a better indication of the function, and less noise, compared to computing the composition from a single sequence.

The impact of incorporating different sources of evolutionary information is presented in Table 4. The compositions computed from a single BLAST search had an average improvement from the baseline of 8.55%. The psi-composition further enhanced the accuracy, with an average improvement from baseline of 10.42%. The improved performance between psi-compositions and BLAST-compositions was expected because, unlike BLAST, which only uses a general scoring matrix, PSI-BLAST uses a position-specific scoring matrix (PSSM) to detect sequences with a similar conservation pattern to the PSSM, thus making PSI-BLAST more sensitive to weak but biologically significant sequence relationships [11].

**Table 4:** Impact of incorporating evolutionary information on the accuracy

Encoding	Accuracy(%)			blast-X to X	psi-X to X	psi-X to blast-X
X	X	blast-X	psi-X	increase(%)	increase(%)	increase(%)
AAC	75.79	84.61	86.13	+ 08.82	+ 10.34	+ 01.52
PAAC	75.41	86.62	87.29	+ 11.21	+ 11.88	+ 00.67
PseAAC	76.19	81.81	85.13	+ 05.62	+ 08.94	+ 03.32
Average	75.80	84.35	86.18	+ 08.55	+ 10.39	+ 01.84

The table notes differences in accuracy and the percentage of improvement when incorporating different evolutionary information to the baseline compositions. The highest improvement in accuracy was achieved by psi-compositions, with an average improvement of 10.39%

### Performance of annotation transfer by homology

The performance of annotation transfer by homology against TCDB under different thresholds is presented in Table 5. The choice of a proper similarity threshold is critical. As shown in Table 5, there is a trade off between sensitivity and specificity, where a stricter threshold (TCDB_exact) results in low true transporter (sensitivity) detection but more reliable elimination of non-transporters (specificity). However, when the thresholds are set to be more tolerant (TCDB_med), the percentage transporter detection increases but at the cost of more false predictions. A good balance between sensitivity and specificity was achieved using thresholds suggested by [3], and the overall accuracy reached 85.72%, slightly lower than the best machine-learning method psiPAAC. Nevertheless, this gives a different solution viewpoint, which we utilize in the ensemble classifier.

**Table 5:** Performance of annotation transfer by homology

	Name	Sensitivity (%)	Specificity (%)	Accuracy (%)	MCC
ATH					
	TCDB_exact	56.92	95.17	73.55	0.5440
	TCDB_high	85.90	85.50	85.72	0.7112
	TCDB_med	90.38	64.17	78.98	0.5737

The table shows the performance homology annotation transfer with the training dataset using different thresholds. The best prediction power was achieved using the TCDB_high threshold. The predicted transporter from TCDB_exact was more reliable due to its high specificity. ATH= Annotation Transfer by Homology

### Ensemble classifiers

The performance of the ensemble classifier, and each of its constituent classifiers in the cross-validation and independent dataset is presented in Tables 6 and 7. The ensemble classifier consistently outperformed its classifiers in detecting transporters (sensitivity) while maintaining a credible false positive rate. Overall, it surpassed all other tested models in terms of accuracy and the MCC.

**Table 6:** Cross-validation performance of the proposed model

	name	Sensitivity (%)	Specificity (%)	Accuracy (%)	MCC
SVM					
	psiAAC	82.69 ±00.21	90.64 ±00.41	86.13 ±00.15	0.7278 ±0.0036
	psiPAAC	86.73 ±00.29	87.99 ±00.54	87.29 ±00.11	0.7448 ±0.0027
	psiPseAAC	80.43 ±00.43	91.47 ±00.46	85.23 ±00.34	0.7142 ±0.0069
ATH					
	TCDB_exact	56.92	95.17	73.55	0.5440
	TCDB_high	85.90	85.50	85.72	0.7112
	TCDB_med	90.38	64.17	78.98	0.5737
Proposed_Ensemble*		90.15 ±00.24	89.97 ±00.34	90.07 ±00.07	0.7995 ±0.001

The table lists the *mean*±*sd* performance of ten different runs of the 10-CV of the proposed ensemble. It also shows the performance of each of its constituent classifiers

^*^The proposed model; ATH = Annotation Transfer by Homology

**Table 7:** Independent testing performance of the proposed model

	name	Sensitivity (%)	Specificity (%)	Accuracy (%)	MCC
SVM					
	psiAAC	83.33	95.00	87.22	0.75
	psiPAAC	89.17	88.33	88.89	0.76
	psiPseAAC	80.00	96.67	85.56	0.73
ATH					
	TCDB_exact	56.67	91.67	68.33	0.46
	TCDB_high	86.67	80.00	84.44	0.66
	TCDB_med	92.5	58.33	81.11	0.56
Proposed_Ensemble*		94.17	88.33	92.22	0.82

The table shows the performance of the proposed ensemble and each of its constituent classifiers

^*^The proposed model; ATH = Annotation Transfer by Homology

It was previously shown by [23, 24] that ensemble classifiers benefited the most when the individual classifiers making up the ensemble were both *accurate* and have *low correlation* (i.e., making errors in different parts of the input space). The constituent classifiers in our ensemble achieved the highest accuracy, and the correlations between them are presented in Table 8. When combining the prediction of only the three models on the machine-learning side, we observed no improvement in overall accuracy. This is reasonable since the machine-learning models in our case were highly correlated. The obtained performance was mainly achieved by combining a different view — annotation transfer by homology, which has comparable accuracy to machine-learning classifiers but lower correlation.

**Table 8:** Pearson correlation coefficient of constituent classifiers

model	psiAAC	psiPAAC	psiPseAAC	TCDB_exact	TCDB_high	TCDB_med
psiAAC	1.00	0.81	0.90	0.56	0.63	0.52
psiPAAC	0.81	1.00	0.80	0.51	0.61	0.50
psiPseAAC	0.90	0.80	1.00	0.55	0.62	0.52
TCDB_exact	0.56	0.51	0.55	1.00	0.65	0.51
TCDB_high	0.63	0.61	0.62	0.65	1.00	0.78
TCDB_med	0.52	0.50	0.52	0.51	0.78	1.00

The table shows the correlation between the constituent classifiers of the ensemble. Among themselves, the homology annotation transfer exhibit a lower correlation compared to those of the machine-learning models. This lower correlation motivates the use of ensemble techniques and helps to build a more powerful model

### Comparative performance of the proposed tool with other published work

Table 9 compares the performance of the proposed model with other published work. The highest prediction accuracy was achieved by Li et al. [8]. The high performance of their model was mainly due to using the Gene Ontology (GO) annotation of the proteins as features. Such high performance is to be expected, considering the fact that all the sequences in the benchmark dataset were well annotated and extracted from the Swiss-Prot database. The goal of TooT-T is to predict novel unannotated transporters proteins.

**Table 9:** Comparison with other published work

*Tool*	Sensitivity(%)		Specificity (%)		Accuracy (%)		MCC	
	Ind.	CV	Ind.	CV	Ind.	CV	Ind.	CV
*SCMMTP* [7]	80.00	83.76	68.33	77.68	76.11	81.12	0.47	0.62
TrSSP [6]	76.67	76.67	81.67	78.46	80.00	78.99	0.57	0.58
Ou et al. [9]	100.00	83.14	77.50	84.48	85.00	83.94	0.73	0.68
Proposed model	94.17	90.15	88.33	89.97	92.22	90.07	0.82	0.80
Li et al. [8]	96.67	99.50	95.83	97.44	96.11	98.33	0.91	0.97

The other tools did not incorporate annotations of proteins as features and relied solely on the protein sequence to extract features to distinguish between transporters and non-transporters. They therefore provide a better comparison with the proposed tool. Ou et al. [9] tool achieved better sensitivity (100%) than the proposed tool (94.17%) in the independent dataset. However, the specificity was (77.50%) compared to (88.33%) obtained by the proposed tool. The proposed tool achieved (7%) higher accuracy, and (0.09) higher MCC than Ou et al. [9] tool in transporter detection. Overall, TooT-T achieved better accuracy, specificity, and MCC than all tools reported in all other published works, both in independent and cross-validation testing.

## Conclusion

We propose an ensemble classifier that can distinguish transporter membrane proteins from other proteins. The ensemble classifier is trained to optimally combine the prediction obtained from machine-learning and homology annotation methods to produce the final prediction. The machine-learning components of the ensemble consist of SVM models that incorporate a novel feature extraction method *psi-composition*. The psi-composition combines traditional amino acid composition with the alignment results of PSI-BLAST and shows superior prediction performance to models built using other features, including the PSSM profile. While the prediction obtained from annotation transfer by homology was not superior to the best machine-learning models, it provided a different viewpoint on the solution. The proposed ensemble exploits the fact that different methods misclassify different sequences to build a more credible model. It was demonstrated through repeated 10-fold cross-validation and independent dataset tests that the proposed ensemble outperformed its constituent classifiers and all other state-of-the art predictors that rely on the protein sequence alone.

## Data Availability

The dataset used in the current study is publicly available at: http://bioinfo.noble.org/TrSSP/?dowhat=Datasets

## Abbreviations

AAC: Amino acid composition
CV: Cross validation
GBM: Gradient boosting machine
GO: Gene ontology
IUBMB: International union of biochemistry and molecular biology
MCC: Matthews correlation coefficient
PAAC: Pair amino acid composition
PseAAC: Pseudo amino acid composition
PSSM: Position-specific scoring matrix
SCM: Scoring card method
SVM: Support-vector machine
TCDB: Transporter classification database
